# Chronic Hemifacial Pain and Temporomandibular Joint Symptoms in a Patient With Posterior Occlusal Collapse Undergoing Implant Rehabilitation: A Case Report

**DOI:** 10.7759/cureus.115395

**Published:** 2026-08-29

**Authors:** Keily J Portillo Avila, Richard M Cavero, Fabiana Lora, Alexandra C Campos Guardia, Rosa V Mota

**Affiliations:** 1 Periodontics, Dental Loft, San Pedro Sula, HND; 2 Dentistry, Vedha Family Dentistry, Dover, USA; 3 Dentistry, Love My Smile Dental, New York, USA; 4 Dentistry, Family Dental Cosmetic Dentistry, Scarsdale, USA; 5 Dentistry, North Dental Care, Fort Laurderdale, USA; 6 Dentistry, Central Florida Cosmetic and Family Dentistry, Kissimmee, USA

**Keywords:** dental implants, hemifacial pain, implant rehabilitation, malocclusion, migraine, occlusal collapse, posterior occlusal support, temporomandibular disorders, temporomandibular joint, tooth loss

## Abstract

Posterior tooth loss may result in loss of occlusal support and alterations in masticatory function; however, the relationship between occlusal factors and temporomandibular disorders (TMDs) is complex, multifactorial, and remains controversial. This report describes the comprehensive rehabilitation of a 38-year-old female patient (American Society of Anesthesiologists (ASA) Physical Status Classification I) who presented with chronic right-sided hemifacial pain, recurrent right-sided headaches, pain localized to the right temporomandibular joint (TMJ), and difficulty chewing. Clinical examination and cone-beam computed tomography (CBCT) demonstrated absence of the mandibular right first and second molars, with loss of posterior occlusal support, together with a mesioangularly impacted mandibular right third molar occupying the posterior edentulous region. Treatment consisted of surgical extraction of the impacted third molar followed by placement of two dental implants and subsequent implant-supported prosthetic rehabilitation of the missing posterior teeth. After a six-month osseointegration period, definitive implant-supported crowns were fabricated using a digital workflow and delivered with careful occlusal adjustment. At the three-month follow-up after prosthetic loading, the patient reported resolution of the chronic hemifacial pain, recurrent headaches, and TMJ discomfort, together with improvement in masticatory function. Clinical and radiographic examinations demonstrated stable peri-implant tissues and satisfactory functional rehabilitation. Because treatment involved sequential surgical and prosthetic interventions and standardized symptom assessments were not obtained after each treatment stage, the improvement cannot be attributed to any individual intervention, nor can a causal relationship between the patient's occlusal condition and presenting symptoms be established. This case documents a temporal association between comprehensive posterior dental rehabilitation and symptom improvement, and further studies are needed to clarify the relationship between posterior occlusal support and temporomandibular symptoms.

## Introduction

Posterior tooth loss is a common clinical condition that can significantly alter occlusal stability and masticatory function. When posterior occlusal support is lost and not restored, progressive tooth migration, changes in occlusal relationships, and alterations in mandibular biomechanics may occur [1-4]. Although these changes may affect masticatory function and chewing efficiency, their relationship with temporomandibular disorders (TMDs) and orofacial pain is complex and remains controversial [5,6].

TMDs represent a heterogeneous and multifactorial group of conditions involving the temporomandibular joint (TMJ), masticatory muscles, and associated structures [7-10]. Patients may present with joint pain, muscle tenderness, limited mandibular function, headaches, or facial pain. Historically, occlusal factors were considered important in the development of TMD; however, contemporary models recognize that TMDs may reflect interactions among biological, biomechanical, psychological, behavioral, and other factors rather than a single occlusal mechanism. Therefore, posterior tooth loss or altered occlusion should not be considered an established cause of TMD or chronic facial pain. Careful clinical examination is important for evaluating the patient's dental and occlusal condition, while cone-beam computed tomography (CBCT) can provide three-dimensional assessment of impacted teeth, edentulous regions, available bone, and adjacent anatomical structures relevant to treatment planning [11-15].

Dental implants are an established treatment option for replacing missing posterior teeth and restoring posterior occlusal support and masticatory function [16-18]. However, improvement in chronic facial pain, headaches, or TMJ symptoms following posterior dental rehabilitation should not be interpreted as evidence of a direct causal relationship, particularly because these symptoms may have multiple potential etiologies and may fluctuate over time.

Although posterior tooth loss and reduced occlusal support have been investigated in relation to masticatory function and temporomandibular symptoms, the clinical relationship between restoration of posterior occlusal support and changes in chronic orofacial symptoms remains incompletely understood and should not be assumed to be causal. This case illustrates the diagnostic and therapeutic complexity encountered when chronic orofacial symptoms coexist with significant posterior dental abnormalities requiring rehabilitation. Reporting the clinical course provides an opportunity to compare the observed outcome with existing evidence while emphasizing the limitations of attributing symptom changes to occlusal or restorative interventions. Hence, this report contributes a clinically documented observation to the literature concerning posterior occlusal rehabilitation and temporomandibular and orofacial symptoms.

## Case presentation

A 38-year-old female patient (American Society of Anesthesiologists (ASA) Physical Status Classification I [19]) with no known drug allergies presented with chronic right-sided hemifacial pain, recurrent right-sided headaches, discomfort localized to the right TMJ, and progressive difficulty chewing, particularly on the right side. The patient reported that these symptoms had persisted for several years. A formal migraine diagnosis based on established headache diagnostic criteria and a specific TMD diagnosis based on the Diagnostic Criteria for Temporomandibular Disorders (DC/TMD) [20] were not documented. Detailed standardized characterization of pain intensity, frequency, episode duration, pain quality, associated symptoms, and aggravating or relieving factors was not available in the clinical record. Therefore, the facial pain, headaches, and TMJ discomfort are reported as presenting symptoms rather than as established headache or TMD diagnoses.

Clinical examination revealed absence of the mandibular right first and second molars (teeth #30 and #31), resulting in loss of posterior occlusal support in this region. A panoramic radiograph demonstrated a mesioangularly impacted mandibular right third molar (tooth #32), positioned at approximately 45° and occupying the posterior edentulous region (Figure 1). No quantitative or computerized occlusal force analysis was performed; therefore, the occlusal findings are reported descriptively rather than as objective evidence of abnormal biomechanical loading. The dental and occlusal findings were considered relevant to treatment planning but were not regarded as establishing the etiology of the patient's chronic hemifacial pain, headaches, or TMJ discomfort.

**Figure 1:**
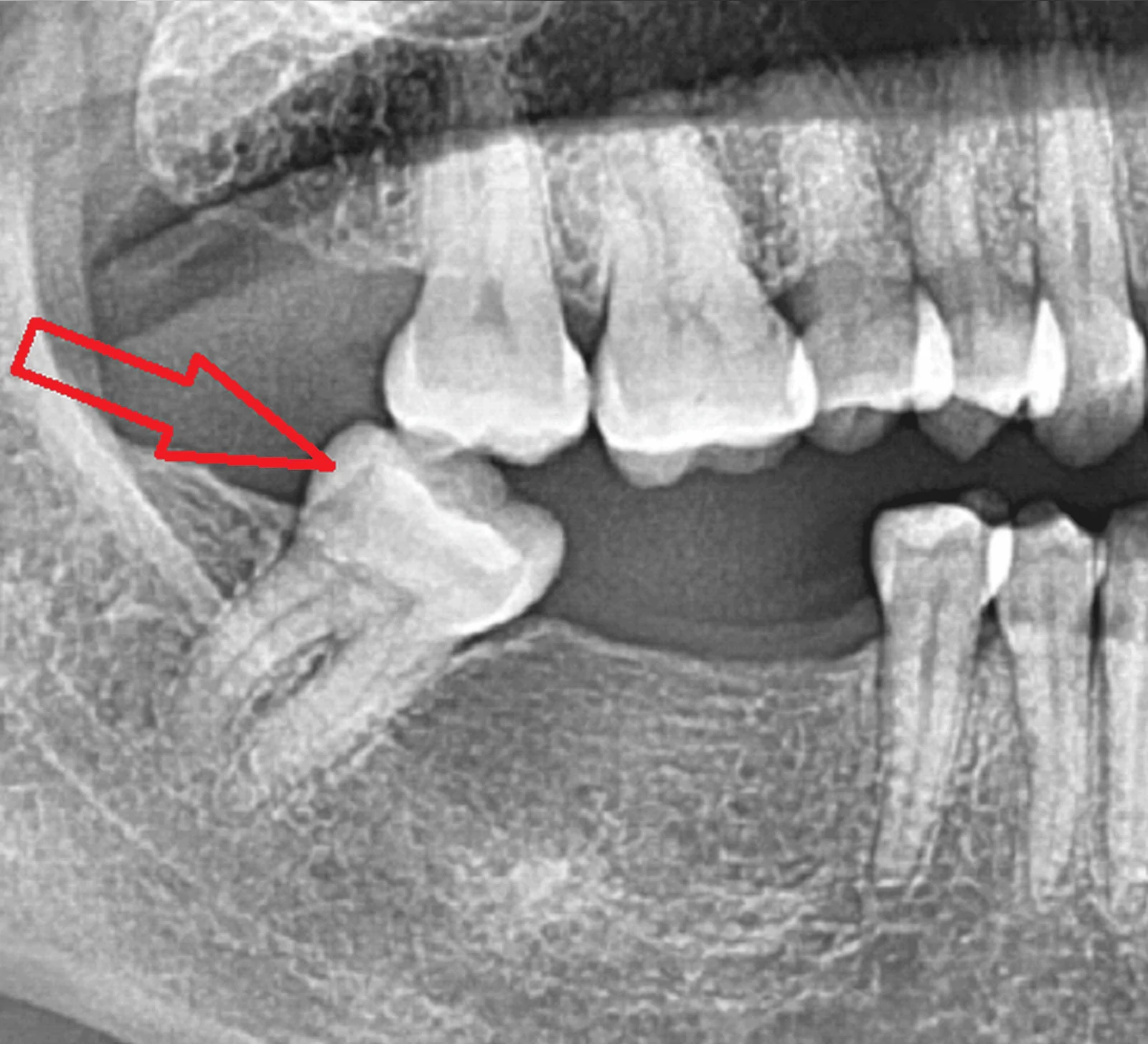
Preoperative panoramic radiograph demonstrating a mesioangularly impacted mandibular right third molar (tooth #32), indicated by red arrow, occupying the posterior mandibular edentulous space. The absence of mandibular right first and second molars (teeth #30 and #31) resulted in loss of posterior occlusal support, while the impacted third molar occupied the edentulous space. These findings documented the posterior dental and occlusal conditions relevant to treatment planning but do not establish the etiology of the patient's chronic right-sided hemifacial pain, headaches, or TMJ discomfort. TMJ: temporomandibular joint

Following discussion of the clinical findings, treatment options, risks, benefits, and alternatives, informed consent was obtained. A comprehensive dental treatment plan was established consisting of surgical extraction of the impacted mandibular right third molar followed by implant-supported rehabilitation of the missing posterior teeth.

Local anesthesia was achieved using 2% lidocaine with 1:100,000 epinephrine. A crestal incision was performed using a No. 15 blade, and a full-thickness mucoperiosteal flap was elevated. The impacted mandibular right third molar was surgically removed, and the implant osteotomies were prepared according to the manufacturer's recommended drilling protocol. Virtual implant planning was performed using CBCT to evaluate the posterior mandibular anatomy and determine prosthetically appropriate implant positions before surgery (Figure 2).

**Figure 2:**
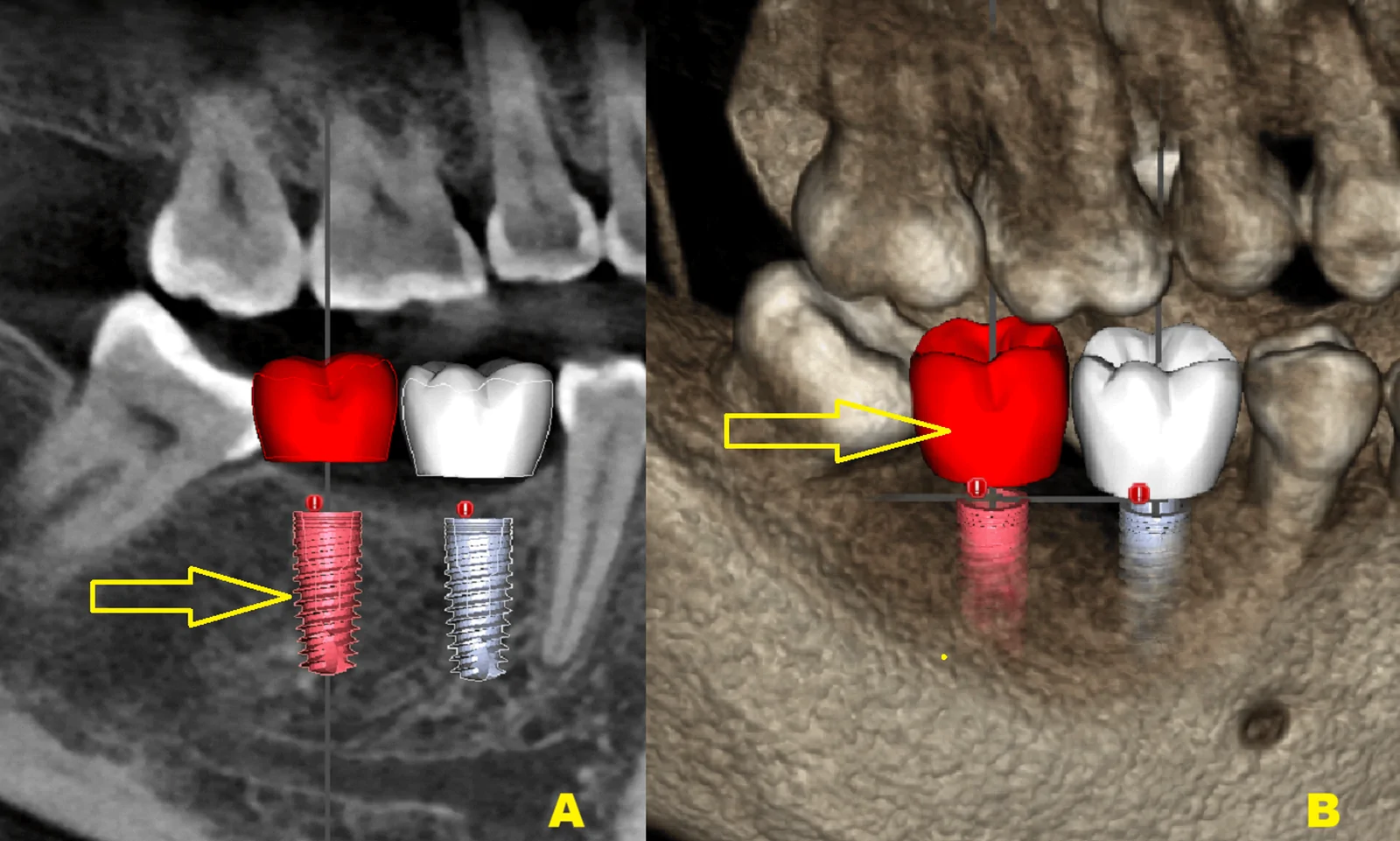
CBCT treatment planning for implant-supported rehabilitation. (A) Cross-sectional CBCT view demonstrating virtual treatment planning for placement of two dental implants in the mandibular right posterior edentulous region corresponding to the missing mandibular right first and second molars (teeth #30 and #31). The yellow arrow indicates the virtually planned implant in the mandibular right first molar position. (B) Three-dimensional CBCT reconstruction illustrating the planned implant-supported crowns in their proposed prosthetically driven positions. The yellow arrow indicates the planned implant-supported restoration in the mandibular right first molar position. CBCT: cone-beam computed tomography

Two wide-diameter dental implants measuring 5.0 × 14 mm were placed in the mandibular right posterior edentulous region with clinically adequate primary stability (Figure 3). Numerical insertion torque, implant stability quotient values, formal bone quality classification, and standardized quantitative peri-implant parameters were not available in the clinical record.

**Figure 3:**
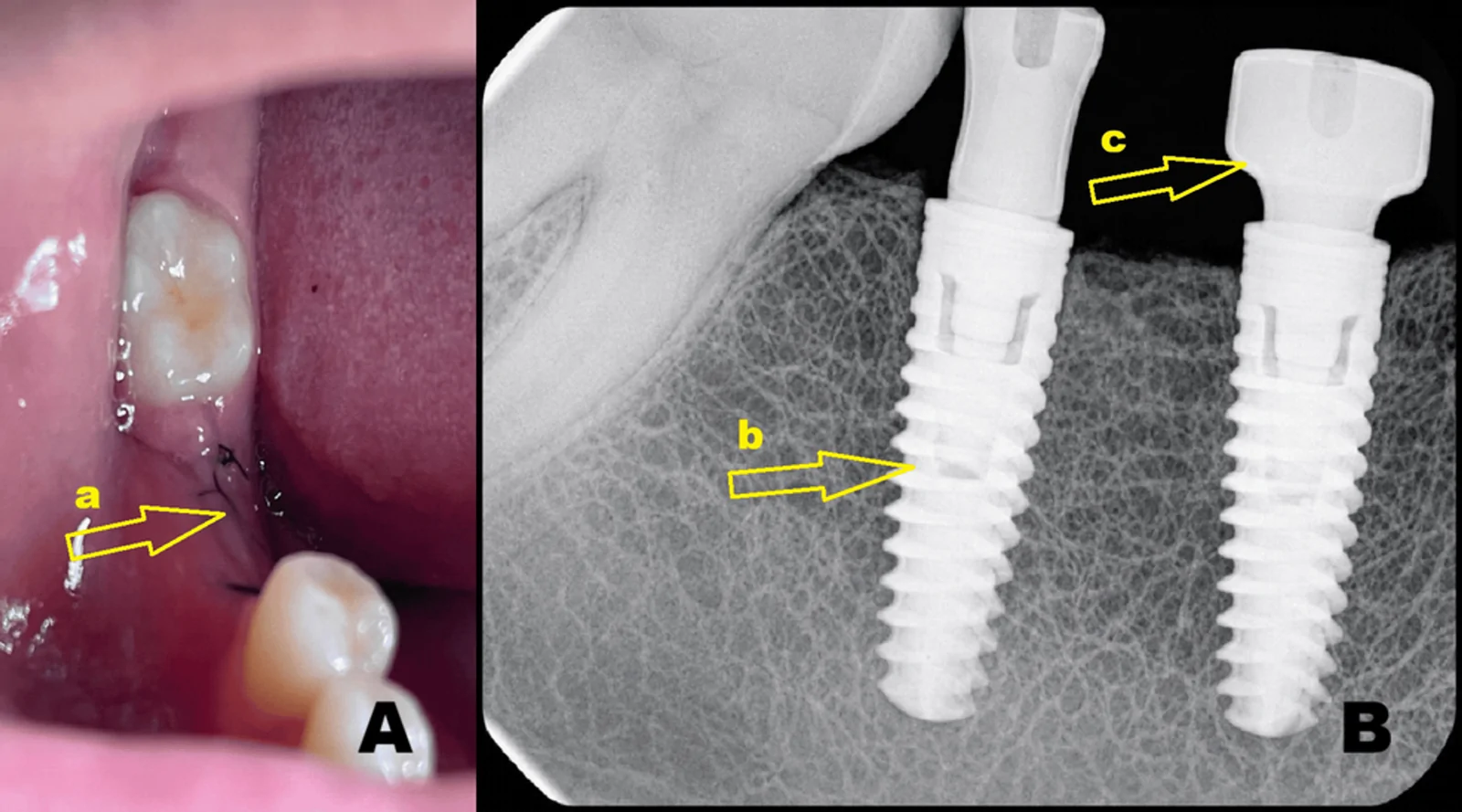
Implant placement and six-month postoperative evaluation prior to definitive restoration. (A) Clinical photograph demonstrating placement of two dental implants in the mandibular right posterior edentulous region immediately after surgery. Yellow arrow (a) indicates the 4-0 nylon sutures used to achieve primary closure. (B) Periapical radiograph obtained after a six-month osseointegration period. Yellow arrow (b) indicates the dental implants, and yellow arrow (c) indicates the scan bodies connected to the implants before digital intraoral scanning for fabrication of the definitive implant-supported restorations.

The surgical sites were thoroughly irrigated, and the flaps were repositioned and closed with interrupted 4-0 nylon sutures to achieve primary closure. Postoperative healing was uneventful. Standardized assessments of the patient's hemifacial pain, headaches, and TMJ discomfort were not recorded after third molar extraction or during the subsequent osseointegration period; therefore, the temporal contribution of the individual surgical and prosthetic interventions to subsequent symptom changes cannot be determined.

After a six-month osseointegration period, scan bodies were connected to the implants, and a digital intraoral scan was obtained for fabrication of the definitive implant-supported restorations. The digital design was used to evaluate the planned implant-supported crowns and their occlusal relationship with the opposing dentition before fabrication (Figure 4).

**Figure 4:**
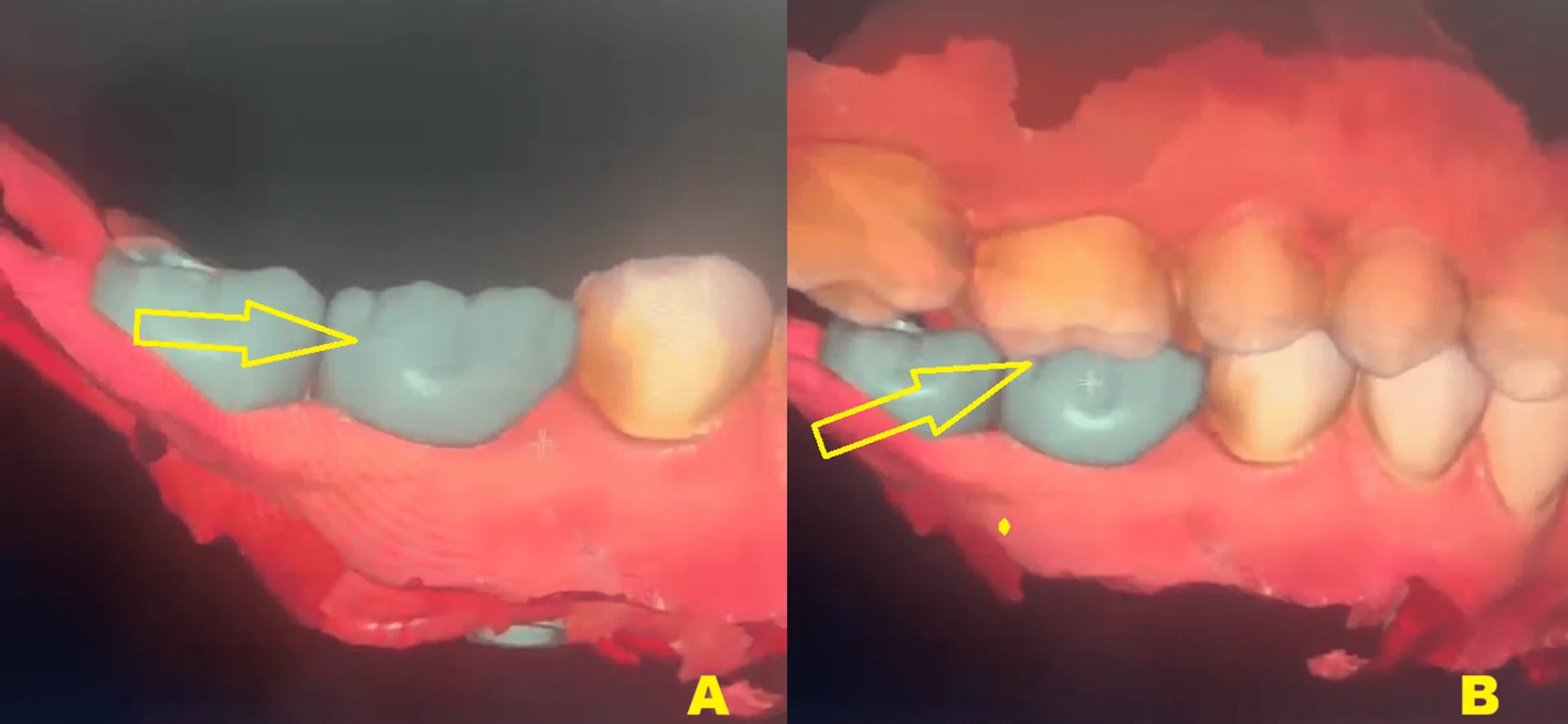
Digital design of the definitive implant supported restorations. (A) Three-dimensional digital planning of the definitive implant supported crowns in the mandibular right posterior region. The yellow arrow indicates the virtual implant supported restorations prior to fabrication. (B) Three-dimensional digital occlusal analysis demonstrating the planned occlusal relationship of the definitive implant supported crowns with the opposing dentition. The yellow arrow indicates the planned occlusal contact of the implant-supported restoration.

The digital records were transferred to the dental laboratory, and three weeks later, the definitive implant-supported crowns replacing teeth #30 and #31 were delivered. Clinical examination demonstrated restoration of posterior occlusal support in the mandibular right posterior region (Figure 5).

**Figure 5:**
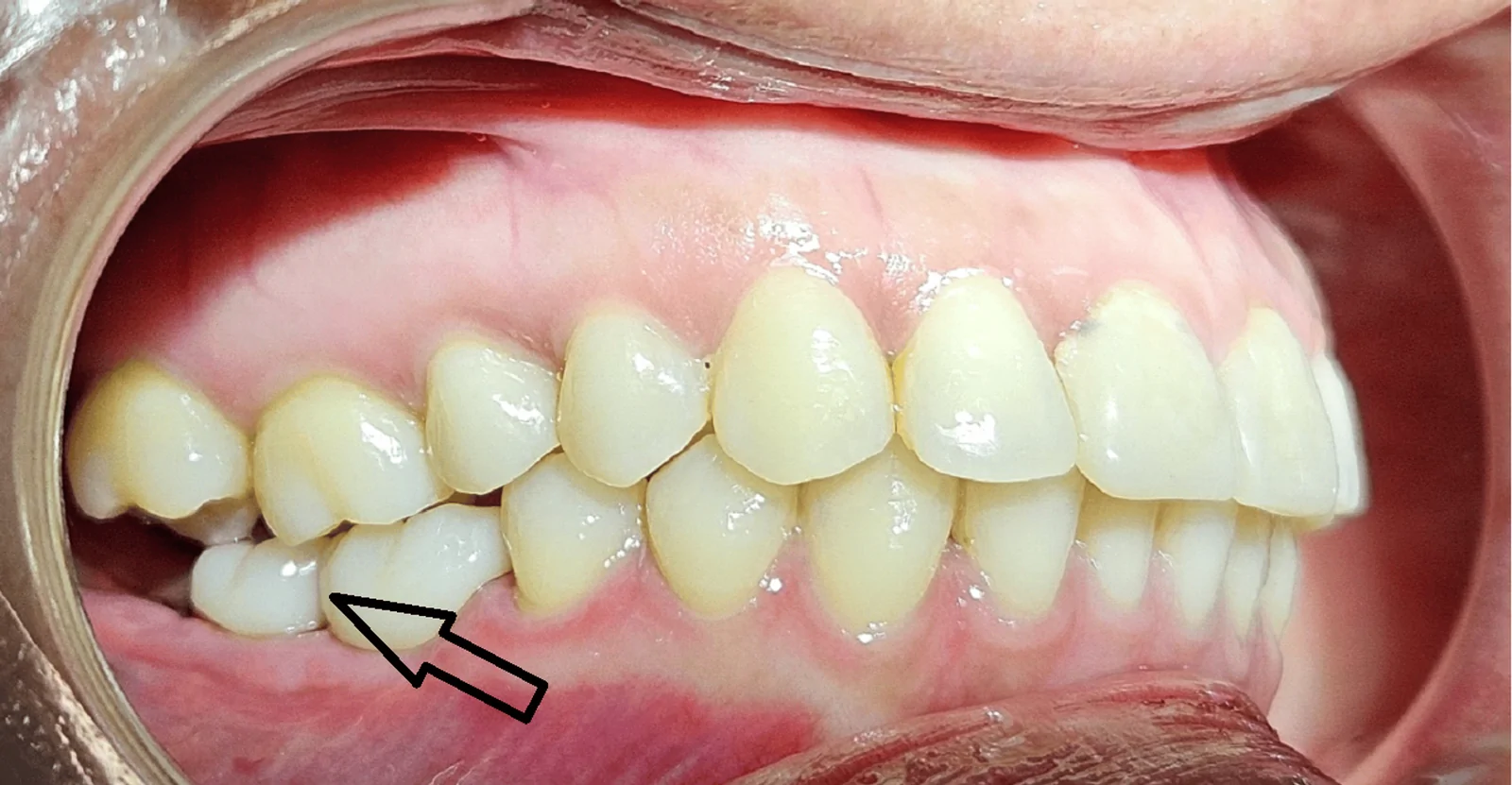
Definitive implant-supported crowns in occlusion. Clinical lateral view obtained three weeks after the digital intraoral scan, demonstrating the definitive implant-supported crowns replacing the mandibular right first and second molars (teeth #30 and #31) in maximum intercuspation. The black arrow indicates the implant supported restorations, showing stable posterior occlusal support and harmonious occlusion with the opposing dentition.

The restorations were clinically evaluated and adjusted to establish appropriate occlusal contact with the opposing dentition in maximum intercuspation. No computerized or quantitative occlusal force analysis was performed.

At the three-month follow-up after prosthetic loading, the patient reported resolution of the previously described chronic right-sided hemifacial pain, recurrent right-sided headaches, and right TMJ discomfort. She also reported improvement in chewing function and overall oral function. These symptomatic outcomes were based on the patient's subjective report; standardized pain scales, headache frequency or disability measures, validated TMD assessments, and quantitative mandibular functional measures were not obtained at baseline or follow-up. Therefore, the observed symptom improvement represents a patient-reported temporal association following sequential dental treatment and cannot establish a causal relationship with any individual component of the treatment.

Clinical examination at the three-month follow-up demonstrated healthy peri-implant soft tissues and clinically stable implant-supported restorations without evident mechanical or biological complications during the observation period. A panoramic radiograph demonstrated the implant-supported crowns replacing teeth #30 and #31 with satisfactory radiographic stability and maintenance of posterior dental rehabilitation (Figure 6).

**Figure 6:**
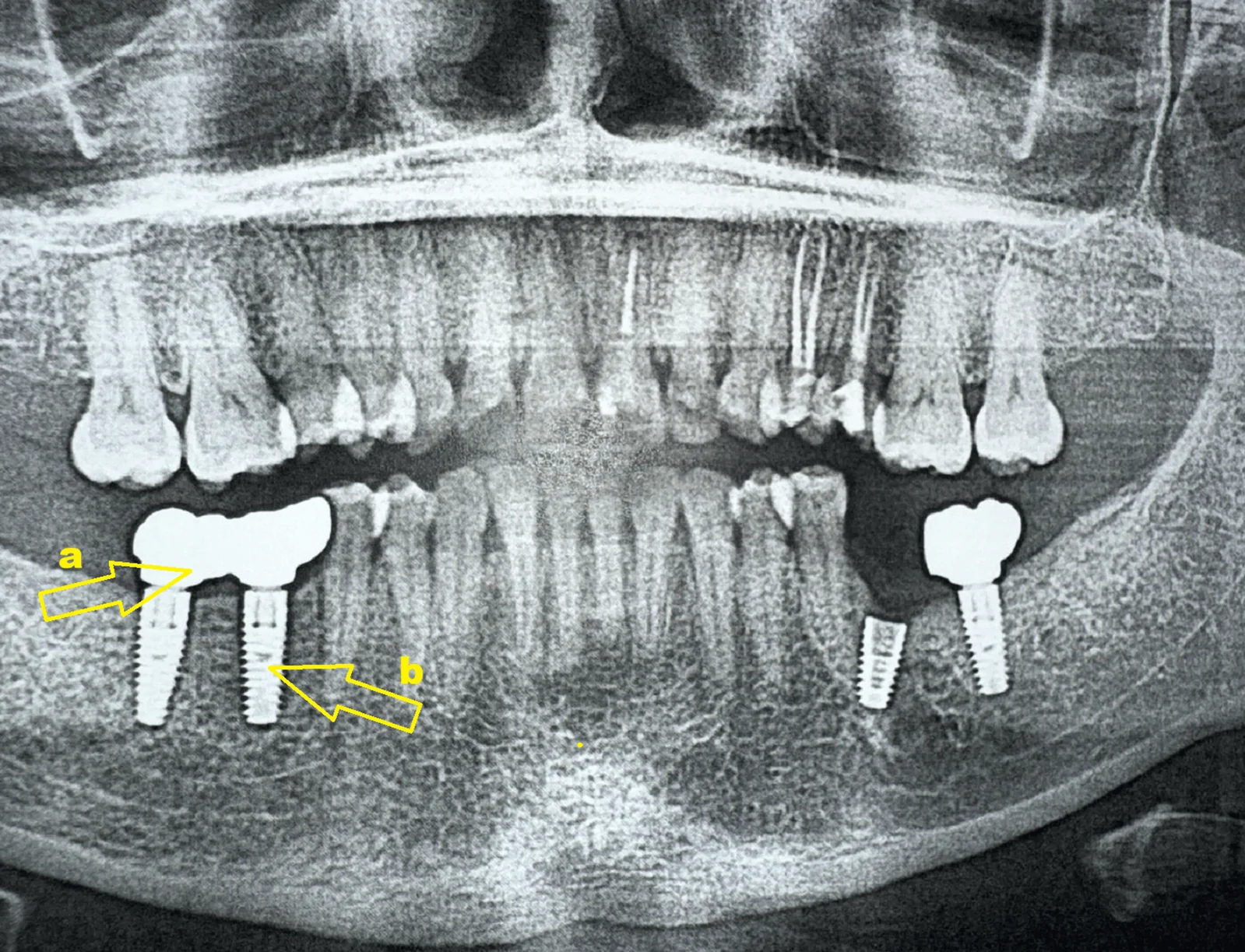
Three-month follow-up panoramic radiograph after prosthetic loading. Panoramic radiograph after delivery of the definitive implant-supported crowns, demonstrating the implant-supported rehabilitation replacing the mandibular right first and second molars (teeth #30 and #31). Yellow arrow (a) indicates the implant supported crowns replacing teeth #30 and #31, while yellow arrow (b) indicates the underlying dental implants. No evident radiographic complications were observed during this short-term follow-up period.

## Discussion

TMD is a heterogeneous and multifactorial condition involving the TMJ, masticatory muscles, and associated structures. Patients may present with jaw pain, facial pain, headaches, joint sounds, or functional limitations. Although occlusal abnormalities have historically been considered potential etiological factors in TMD, contemporary evidence supports a multifactorial model involving biological, biomechanical, psychological, behavioral, and other factors [1-4]. This perspective is particularly relevant to the present case because posterior tooth loss and chronic orofacial symptoms occurred concurrently, but their coexistence does not establish a causal relationship. The patient's missing posterior teeth were therefore considered clinically relevant dental findings rather than evidence that altered occlusion was responsible for the hemifacial pain, headaches, or TMJ discomfort.

Posterior tooth loss may compromise posterior occlusal support and masticatory function. Shifman et al. described the clinical implications of posterior bite collapse [5], while Ikebe et al. reported differences in masticatory performance associated with varying degrees of tooth loss [6]. The present case is consistent with these observations because the patient was missing the mandibular right first and second molars and reported progressive difficulty chewing, particularly on the affected side. However, the case differs in the additional presence of chronic hemifacial pain, headaches, and TMJ discomfort. The available evidence regarding posterior tooth loss and masticatory performance does not establish that these pain symptoms resulted from the missing teeth; therefore, the improvement in chewing function after rehabilitation can be interpreted more directly than the reported improvement in pain-related symptoms.

Dental implants are an established treatment modality for replacing missing teeth and restoring oral function, and implant-supported prostheses have also been associated with improvements in oral health-related quality of life [7-9]. In the present patient, implant-supported rehabilitation restored posterior dental support in the mandibular right region, and the patient subsequently reported improved chewing function. This functional improvement is consistent with the objectives and outcomes described in the implant literature. In contrast, the reported resolution of hemifacial pain, headaches, and TMJ discomfort extends beyond the principal functional outcomes addressed in these studies. Consequently, these symptom changes should not be interpreted as evidence that implant-supported rehabilitation is a treatment for TMD, headache, or chronic facial pain.

The impacted mandibular third molar represented an additional clinical factor. Previous literature has described pathologies associated with mandibular third molar impaction and contemporary indications and approaches for surgical management [10-12]. In this case, the mesioangularly impacted mandibular right third molar occupied the posterior edentulous region and was surgically removed as part of the planned rehabilitation. Unlike reports focused primarily on the pathology or surgical management of impacted third molars, the present case involved sequential extraction followed by implant-supported replacement of the adjacent missing posterior teeth. Because standardized symptom assessments were not performed after third molar extraction and before implant-supported rehabilitation, it cannot be determined whether removal of the impacted tooth contributed independently to the subsequent symptom improvement.

Biomechanical studies provide additional context regarding posterior occlusal support. Sakai and Yamashita demonstrated three-dimensional condylar displacement following simulated loss of posterior occlusal support, while Seedorf et al. reported an influence of posterior occlusal support on condylar position [13,16]. These findings suggest that changes in posterior support can influence mandibular biomechanics under the studied conditions. However, the present case differs because no quantitative occlusal analysis, condylar position measurement, or computerized assessment of force distribution was performed. Therefore, although a biomechanical effect of posterior support is plausible, it cannot be demonstrated in this patient and should not be used to explain the reported resolution of symptoms.

Population-based studies have also examined the dental consequences of reduced posterior occlusal support. Mameno et al. reported an association between posterior occlusal support and tooth loss [14], while Fushida et al. found that decreasing posterior occlusal support area could accelerate tooth loss [15]. These findings reinforce the importance of posterior support from a dental and functional perspective. However, these studies focused on dental outcomes rather than demonstrating that restoration of posterior support resolves chronic facial pain or TMD symptoms. The present case is therefore consistent with the literature regarding the clinical importance of posterior dental support, while the accompanying improvement in hemifacial pain, headaches, and TMJ discomfort remains a separate patient-reported observation.

CBCT is useful in implant dentistry for three-dimensional evaluation of osseous anatomy and adjacent anatomical structures and can assist in implant treatment planning [17,18]. In the present case, CBCT was used to evaluate the posterior mandibular anatomy and determine prosthetically appropriate implant positions. Its role was therefore consistent with established applications of CBCT in implant dentistry. Importantly, CBCT was used for anatomical assessment and treatment planning and not to establish the etiology of the patient's chronic hemifacial pain, headaches, or TMJ discomfort.

An important limitation is the sequential nature of the treatment. Standardized symptom assessments were not documented after extraction of the impacted third molar, during the subsequent six-month osseointegration period, or immediately before prosthetic loading. Consequently, the relative contributions of third molar extraction, implant placement, definitive prosthetic rehabilitation, natural fluctuation of chronic pain symptoms, or other unmeasured factors to the reported improvement cannot be determined. Similarly, although restoration of posterior occlusal support may theoretically influence mandibular biomechanics, force distribution and functional loading were not objectively measured in this case; therefore, such mechanisms remain hypothetical.

Additional limitations include the single case design and the relatively short three-month follow-up after definitive prosthetic loading. Validated pain scales, headache frequency or disability measures, standardized DC/TMD assessments [20], quantitative jaw function or chewing assessments, electromyographic analysis, and validated quality of life instruments were not obtained. Furthermore, chronic facial pain and temporomandibular symptoms may fluctuate naturally over time. Longer follow-up, ideally 6-12 months or more, would be necessary to determine whether the patient-reported symptom improvement persists. These limitations preclude causal interpretation and limit the generalizability of the findings.

The value of this case lies in documenting the clinical course of a patient with chronic unilateral facial symptoms, loss of mandibular right posterior occlusal support, and an impacted mandibular third molar, who underwent sequential surgical and implant-supported dental rehabilitation and subsequently reported symptom improvement. Comparison with the existing literature demonstrates consistency with previously reported functional consequences of posterior tooth loss and the established role of implant-supported rehabilitation in restoring oral function, while the reported improvement in chronic orofacial symptoms represents a notable difference that cannot be attributed to treatment causally. These observations are therefore hypothesis-generating and should not be interpreted as evidence that posterior tooth loss or altered occlusion caused the presenting symptoms or that implant-supported rehabilitation was responsible for their resolution. Prospective studies incorporating standardized diagnostic criteria, validated pain and functional outcome measures, objective occlusal assessments, and longer follow-up are needed to investigate potential relationships between posterior dental rehabilitation and orofacial symptoms.

## Conclusions

In this case, patient-reported improvement in chronic right-sided hemifacial pain, recurrent right-sided headaches, right TMJ discomfort, and chewing function was observed following sequential surgical and implant-supported rehabilitation of the mandibular right posterior region. The case highlights the importance of comprehensive evaluation and restoration of posterior dental function when significant posterior tooth loss and other dental abnormalities coexist with chronic orofacial symptoms, while recognizing that such symptoms may be multifactorial. Because standardized symptom assessments were not performed at each treatment stage, the contributions of third molar extraction, implant placement, prosthetic rehabilitation, natural symptom fluctuation, or other factors cannot be determined. Therefore, the findings represent a temporal clinical association and should not be interpreted as evidence that the patient's posterior occlusal condition caused the presenting symptoms or that implant-supported rehabilitation was responsible for their resolution. The short three-month follow-up after prosthetic loading further limits interpretation of the persistence of the reported improvement. This case may help clinicians recognize the need to distinguish restoration of dental function from treatment of chronic orofacial pain and to avoid attributing symptom improvement to occlusal or restorative interventions without appropriate diagnostic and outcome measures. Prospective studies incorporating standardized diagnostic criteria, validated pain and functional outcome measures, objective occlusal assessments, and longer follow-up are needed to clarify potential relationships between posterior dental rehabilitation and orofacial symptoms.
